# Flipping operators and locally harmonic Maass forms

**DOI:** 10.1007/s11139-025-01183-7

**Published:** 2025-08-08

**Authors:** Kathrin Bringmann, Andreas Mono, Larry Rolen

**Affiliations:** 1 Department of Mathematics and Computer Science, University of Cologne, Weyertal 86-90, 50931 Cologne, Germany; 2 Department of Mathematics, Vanderbilt University, 1326 Stevenson Center, Nashville, TN 37240 USA

**Keywords:** Eisenstein series, Flipping operator, Harmonic maass forms, Integral binary quadratic forms, Modular forms, Poincaré series, 11F03, 11F11, 11F25, 11F37

## Abstract

In the theory of integral weight harmonic Maass forms of manageable growth, two key differential operators, the Bol operator and the shadow operator, play a fundamental role. Harmonic Maass forms of manageable growth canonically split into two parts, and each operator controls one of these parts. A third operator, called the flipping operator, exchanges the role of these two parts. Maass–Poincaré series (of parabolic type) form a convenient basis of negative weight harmonic Maass forms of manageable growth, and flipping has the effect of negating an index. Recently, there has been much interest in locally harmonic Maass forms defined by the first author, Kane, and Kohnen. These are lifts of Poincaré series of hyperbolic type, and are intimately related to the Shimura and Shintani lifts. In this note, we prove that a similar property holds for the flipping operator applied to these Poincaré series.

## Introduction and statement of results

Throughout, we let $k\in \mathbb {N}_{\ge 2}$. The *flipping operator* is defined as (see [4])

$$\begin{aligned} {\mathfrak {F}}_{2-2k}(f)(\tau ) {:}{=}-\frac{v^{2k-2}}{(2k-2)!} \overline{R_{2-2k}^{2k-2}(f)(\tau )}, \end{aligned}$$

where $\tau =u+iv \in \mathbb {H}:=\{\tau \in \mathbb {C}:v>0\}$, the complex upper half-plane, throughout. Here, for $\kappa \in \mathbb {Z}$, the *(iterated) Maass raising operator* is given as ($n \in \mathbb {N}$)

$$\begin{aligned} R_{\kappa } {:}{=}2i\frac{d}{d\tau } + \frac{\kappa }{v}, \quad R_{\kappa }^n {:}{=}R_{\kappa +2(n-1)} \circ \ldots \circ R_{\kappa }. \end{aligned}$$

The goal of this paper is to show that the flipping operator keeps a certain quadratic form Poincaré series invariant up to sign.

The flipping operator acts on the weight $2-2k$ Maass–Poincaré series ${\mathcal {P}}_{2-2k,m}$ of index $m \in \mathbb {Z}\setminus \{0\}$ defined in (2.2) by (see [2, Theorem 6.11 iv)])

$$\begin{aligned} {\mathfrak {F}}_{2-2k}({\mathcal {P}}_{2-2k,m}) = {\mathcal {P}}_{2-2k,-m}. \end{aligned}$$

This “flipping” of *m* and $-m$ is actually where the name “flipping operator” comes from. For the Maass–Eisenstein series ${\mathcal {E}}_{2k}$ defined in (2.3), we have (see [2, Theorem 6.15 iv)])

1.1 $$\begin{aligned} {\mathfrak {F}}_{2-2k}({\mathcal {E}}_{2k}) = -{\mathcal {E}}_{2k}. \end{aligned}$$

The flipping operator is closely related to the *Bol operator* [7] and the *shadow operator* [6], which are respectively given by

$$\begin{aligned} {\mathcal {D}}^{2k-1} {:}{=}\left( \frac{1}{2\pi i} \frac{\partial }{\partial \tau } \right) ^{2k-1}, \qquad \xi _{2-2k} {:}{=}2iv^{2-2k} \overline{\frac{\partial }{\partial \overline{\tau }}}. \end{aligned}$$

Let $P_{2k,m}$ and $E_{2k}$ be the usual (weakly) holomorphic Poincaré and Eisenstein series, respectively, defined in (2.1). Then (see [2, Theorem 6.11 ii), iii))], we have

$$\begin{aligned} \xi _{2-2k} ({\mathcal {P}}_{2-2k,m}) = -\frac{(4\pi m)^{2k-1}}{(2k-2)!} P_{2k,-m}, \qquad {\mathcal {D}}^{2k-1} ({\mathcal {P}}_{2-2k,m}) = m^{2k-1} P_{2k,m}. \end{aligned}$$

For the Maass–Eisenstein series ${\mathcal {E}}_{2-2k}$ (see [2, Theorem 6.15 ii), iii)]), we have

1.2 $$\begin{aligned} \xi _{2-2k} ({\mathcal {E}}_{2-2k}) = (2k-1) E_{2k}, \quad {\mathcal {D}}^{2k-1} ({\mathcal {E}}_{2-2k}) = -(4\pi )^{1-2k}(2k-1)! E_{2k}. \end{aligned}$$

We refer to (2.8) and (2.9) for the interplay between ${\mathfrak {F}}_{2-2k}$, $\xi _{2-2k}$, and ${\mathcal {D}}^{2k-1}$.

The aim of this paper is to show that (1.1) extends to a certain quadratic form Poincaré series. To describe this, we let $D \in \mathbb {N}$ be a non-square discriminant, and ${\mathcal {Q}}_D$ be the set of integral binary quadratic forms $Q(x,y) {:}{=}[a,b,c](x,y) {:}{=}ax^2+bxy+cy^2$ of discriminant $D {:}{=}b^2-4ac$ throughout. Zagier [20] defined

$$\begin{aligned} f_{k,D}(\tau ) {:}{=}\frac{D^{k-\frac{1}{2}}}{\left( {\begin{array}{c}2k-2\\ k-1\end{array}}\right) \pi } \sum _{Q \in {\mathcal {Q}}_{D}} \frac{1}{Q(\tau ,1)^k} \in S_{2k}, \end{aligned}$$

where $S_{2k}$ denotes the space of weight 2*k* cusp forms for $\textrm{SL}_2(\mathbb {Z})$. These functions have very fruitful applications in the theory of modular forms [13, 16, 17]. Katok [14] showed that one can write $f_{k,D}$ as a hyperbolic Eisenstein series (see [12] for more details on hyperbolic expansions), and used this to prove that the $f_{k,D}$ generate $S_{2k}$ as *D* ranges over positive discriminants. Paralleling the behavior of the parabolic Eisenstein series in (1.2), the first author, Kane, and Kohnen [3] discovered a weight $2-2k$ preimage ${\mathcal {F}}_{1-k,D}$ of $f_{k,D}$ (up to constants) under both the Bol operator and the shadow operator. To introduce it, we define for $Q=[a,b,c] \in {\mathcal {Q}}_D$

$$\begin{aligned} Q_{\tau } {:}{=}\frac{1}{v}\left( a\left( u^2+v^2\right) +bu+c\right) , \qquad \beta (x;r,s) {:}{=}\int _{0}^{x} t^{r-1} (1-t)^{s-1} dt \end{aligned}$$

for $x \in [0,1]$ and $r,s > 0$. Then the function ${\mathcal {F}}_{1-k,D}$ is given by (see [3, (1.4)])

1.3 $$\begin{aligned} {\mathcal {F}}_{1-k,D}(\tau ) {:}{=}\! \frac{D^{\frac{1}{2}-k}}{2\left( {\begin{array}{c}2k-2\\ k-1\end{array}}\right) \pi } \sum _{Q \in {\mathcal {Q}}_D} {{\,\textrm{sgn}\,}}(Q_{\tau })Q(\tau ,1)^{k-1} \beta \!\left( \frac{Dv^2}{\left|Q(\tau ,1) \right|^2}; k\!-\!\frac{1}{2}, \frac{1}{2}\right) \!.\qquad \end{aligned}$$

Analogously to $f_{k,D}$, one may view ${\mathcal {F}}_{1-k,D}$ as a hyperbolic Eisenstein series of negative weight. Paralleling (1.2), it was shown in [3, Theorem 1.2],

1.4 $$\begin{aligned} \xi _{2-2k}({\mathcal {F}}_{1-k,D}(\tau )) = D^{\frac{1}{2}-k} f_{k,D}(\tau ), \quad {\mathcal {D}}^{2k-1}({\mathcal {F}}_{1-k,D}(\tau )) = -\frac{(2k-2)!}{(4\pi )^{2k-1}} D^{\frac{1}{2}-k} f_{k,D}(\tau )\qquad \end{aligned}$$

outside a certain “exceptional set” $E_D \subseteq \mathbb {H}$ (see (2.10)) of measure^1^ 0. Thus, it is natural to ask if (1.1) extends from a parabolic to a hyperbolic setting as well. We show that this is indeed the case.

### Theorem 1.1

If $D \in \mathbb {N}$ is a non-square discriminant, $k\ge 2$, and $\tau \not \in E_D$, then we have

$$\begin{aligned} {\mathfrak {F}}_{2-2k}({\mathcal {F}}_{1-k,D}(\tau )) = -{\mathcal {F}}_{1-k,D}(\tau ). \end{aligned}$$

### [Style3 Style3]Remark

In [10, 18], the function ${\mathcal {F}}_{1-k,D}$ played a crucial role to characterize non-trivial vanishing of twisted central *L*-values. More precisely, the main result of both papers is that a twisted central *L*-value of a newform vanishes if and only if an associated “local polynomial” (see (2.11)) is constant. Theorem 1.1 might help to simplify the methods developed in [10, 18] by detecting the vanishing of the non-constant part of that local polynomial.

In [5], the first two authors introduced and investigated the weight $-2k$ variant

$$\begin{aligned} {\mathcal {G}}_{-k,D}(\tau ) {:}{=}\! \frac{1}{2} \sum _{Q \in {\mathcal {Q}}_D} Q(\tau ,1)^{k} \beta \!\left( \frac{Dv^2}{\left|Q(\tau ,1) \right|^2}; k\!+\!\frac{1}{2}, \frac{1}{2}\right) \! \end{aligned}$$

of ${\mathcal {F}}_{1-k,D}$, which has continuously but not differentially removable singularities on $E_D$ (see Lemma 2.5). One may view ${\mathcal {G}}_{-k,D}$ as an “even” analog of ${\mathcal {F}}_{1-k,D}$. Hence, it is natural to expect that Theorem 1.1 extends to ${\mathcal {G}}_{-k,D}$. This is indeed the case, as the following corollary shows.

### Corollary 1.2

If $D \in \mathbb {N}$ is a non-square discriminant, $k\ge 2$, and $\tau \not \in E_D$, then we have

$$\begin{aligned} {\mathfrak {F}}_{-2k}({\mathcal {G}}_{-k,D}(\tau )) = -{\mathcal {G}}_{-k,D}(\tau ). \end{aligned}$$

The paper is organized as follows. In Sect. 2 we review the necessary preliminaries on modular forms, harmonic Maass forms, and locally harmonic Maass forms. Section 3 is devoted to the proofs of Theorem 1.1 and of Corollary 1.2.

## Preliminaries

We summarize some standard notions from the theory of holomorphic modular forms and harmonic Maass forms. We refer to [2] for more details on such forms. More details on locally harmonic Maass forms can be found in [3].

### Poincaré series

For a function $f:{\mathbb {H}}\rightarrow \mathbb {C}$, $\kappa \in \mathbb {Z}$, and a matrix , define the *Petersson slash operator*

$$\begin{aligned} f\big \vert _{\kappa }\begin{pmatrix} a &  b \\ c &  d\end{pmatrix}(\tau ) {:}{=}(c\tau +d)^{-\kappa } f\left( \frac{a\tau +b}{c\tau +d}\right) . \end{aligned}$$

For completeness, we define the variants of modular forms we need.

#### Definition 2.1

Let $f :\mathbb {H}\rightarrow \mathbb {C}$ be a function and $\kappa \in \mathbb {Z}$.

1. We say *f* is a *holomorphic modular form* of weight $\kappa$ if the following hold:
  (i) For $\gamma \in \Gamma$ we have $f\vert _{\kappa }\gamma = f$,
  (ii) *f* is holomorphic on $\mathbb {H}$,
  (iii) *f* is holomorphic at $i\infty$.
  We denote the vector space of functions satisfying these conditions by $M_{\kappa }$.
2. If in addition *f* vanishes at $i\infty$, then we call *f* a *cusp form*. The space of cusp forms is denoted by $S_{\kappa }$.
3. If *f* satisfies the conditions (i) and (ii) from (1) and is permitted to have a pole at $i\infty$, then we call *f* a *weakly holomorphic modular form* of weight ${\kappa }$. The vector space of such functions is denoted by $M_{\kappa }^!$.

To define examples, for $k\in \mathbb {N}_{\ge 2}$ and $m \in \mathbb {Z}$, set

2.1 $$\begin{aligned} P_{2k,m} {:}{=}\sum _{\gamma \in \Gamma _{\infty } \backslash \Gamma } \varphi _m \big \vert _{2k}\gamma , \qquad E_{2k} {:}{=}P_{2k,0}, \end{aligned}$$

where $\varphi _m(\tau ){:}{=}q^m$ with $q {:}{=}e^{2\pi i \tau }$ and . We have

$$\begin{aligned} P_{2k,m} \in {\left\{ \begin{array}{ll} S_{2k} & \text {if } m > 0, \\ M_{2k} & \text {if } m = 0, \\ M_{2k}^! & \text {if } m < 0, \end{array}\right. } \end{aligned}$$

and $P_{2k,m}$ spans $S_{2k}$ resp. $M_{2k}^!$ for $m \ne 0$ (see [2, Theorems 6.8 and 6.9]).

In negative weights, we require the following non-holomorphic modular forms.

#### Definition 2.2

Let $f :\mathbb {H}\rightarrow \mathbb {C}$ be a smooth function.

1. We say *f* is a weight $2-2k$ *harmonic Maass form* if the following hold:
  (i) For $\gamma \in \Gamma$ we have $f\vert _{2-2k}\gamma = f$.
  (ii) We have $\Delta _{2-2k}(f) = 0,$ where the weight $\kappa$ *hyperbolic Laplacian* is defined as
    $$\begin{aligned} \Delta _{2-2k} {:}{=}-v^2\left( \frac{\partial ^2}{\partial u^2}+\frac{\partial ^2}{\partial v^2}\right) + i(2-2k) v\left( \frac{\partial }{\partial u} + i\frac{\partial }{\partial v}\right) . \end{aligned}$$
  (iii) There exists a polynomial $P_f(\tau )\in \mathbb {C}[q^{-1}]$, the *principal part of f* such that, as $v \rightarrow \infty$,
    $$\begin{aligned} f(\tau ) - P_f(\tau ) = O\left( e^{-\varepsilon v}\right) \end{aligned}$$
    for some $$\varepsilon > 0$$.
  We denote the vector space of such functions by $H_{\kappa }$.
2. If *f* satisfies the conditions (i) and (ii) from (1) as well as
  $$\begin{aligned} f(\tau ) = O\left( e^{\varepsilon v}\right) \ \text {as} \ v \rightarrow \infty \ \text {for some} \ \varepsilon > 0, \end{aligned}$$
  then we call *f* a *harmonic Maass form of manageable growth*. We denote the vector space of such forms by $H_{\kappa }^!$.

Let $M_{\mu ,\nu }$ be the *M*-Whittaker function (see [1, Subsection 13.14] for example) and define the *Maass–Poincaré series* (see [2, Definition 6.10])

2.2 $$\begin{aligned} {\mathcal {P}}_{2-2k,m} {:}{=}\sum _{\gamma \in \Gamma _{\infty } \backslash \Gamma } \phi _{2-2k,m}\big \vert _{2-2k} \gamma , \qquad m \in \mathbb {Z}\setminus \{0\}, \end{aligned}$$

where

$$\begin{aligned} \phi _{2-2k,m}(\tau ) {:}{=}\frac{(-{{\,\textrm{sgn}\,}}(m))^{2k-1} (4 \pi |m| v)^{k-1}}{(2k-1)!} M_{{{\,\textrm{sgn}\,}}(m)(1-k),k-\frac{1}{2}}(4 \pi |m| v) e^{2 \pi i m u}. \end{aligned}$$

The *Maass–Eisenstein series* is given by (see [2, p. 104])

2.3 $$\begin{aligned} {\mathcal {E}}_{2-2k} {:}{=}\sum _{\gamma \in \Gamma _{\infty } \backslash \Gamma } v^{2k-1}\big \vert _{2-2k} \gamma . \end{aligned}$$

We have ${\mathcal {E}}_{2-2k} \in H_{2-2k}^!$ and

$$\begin{aligned} {\mathcal {P}}_{2-2k,m} \in {\left\{ \begin{array}{ll} H_{2-2k}^! & \text {if } m > 0, \\ H_{2-2k} & \text {if } m < 0. \end{array}\right. } \end{aligned}$$

By virtue of their Fourier expansion (see [2, Theorem 6.11 v)]), ${\mathcal {P}}_{2-2k,m}$ has a prescribed principal part for $m < 0$ resp. a prescribed non-holomorphic part for $m > 0$ (see (2.6) resp. (2.7) below). For this reason, ${\mathcal {P}}_{2-2k,m}$ span $H_{2-2k}$ (resp. $H_{2-2k}^!$ if restricting to $m > 0$). We refer the reader to [2, Theorems 6.11 and 6.15] for proofs including (1.1), (1.2) and more details.

### Differential operators and Fourier expansions

Let $\gamma \in \textrm{SL}_2(\mathbb {R})$ and $f :\mathbb {H}\rightarrow \mathbb {C}$ be smooth. Then, the Maass raising operator satisfies (see [9, Lemma 2.1.1])

2.4 $$\begin{aligned} R_{\kappa } \left( f \vert _{\kappa } \gamma \right) = R_{\kappa }(f) \vert _{\kappa +2}\gamma . \end{aligned}$$

Moreover, if *f* is an eigenfunction of $\Delta _{\kappa }$ with eigenvalue $\lambda$, then $R_{\kappa }(f)$ is an eigenfunction of $\Delta _{\kappa +2}$ with eigenvalue $\lambda +\kappa$, see [2, Lemma 5.2]. Furthermore, according to [8, (56)], the iterated Maass raising operator and the Bol operator are related by

2.5 $$\begin{aligned} R_{2-2k}^n = \sum _{r=0}^n (-1)^r \left( {\begin{array}{c}n\\ r\end{array}}\right) (2-2k+r)_{n-r} v^{r-n} (4\pi )^r {\mathcal {D}}^r, \end{aligned}$$

where the *rising factorial* is defined as

$$\begin{aligned} (a)_n{:}{=}a(a+1)\cdots (a+n-1), \qquad n \in \mathbb {N}_0. \end{aligned}$$

Bruinier and Funke [6] showed that the Fourier expansion of a harmonic Maass form of manageable growth^2^$f \in H_{2-2k}^{!}$ naturally splits into a holomorphic part and a non-holomorphic part. Namely (see [2, Lemma 4.3]), we have a Fourier expansion of the shape

2.6 $$\begin{aligned} f(\tau ) = \sum _{n \gg -\infty } c_f^+(n) q^n + c_f^-(0)v^{2k-1} + \sum _{\begin{array}{c} n \ll \infty \\ n \ne 0 \end{array}} c_f^-(n)\Gamma (2k-1,-4\pi nv)q^n. \end{aligned}$$

Here, the non-holomorphic part involves the *incomplete Gamma function*

$$\begin{aligned} \Gamma (s,x) {:}{=}\int _x^{\infty } t^{s-1}e^{-t} dt, \end{aligned}$$

defined for ${{\,\textrm{Re}\,}}(s) > 0$ and $x \in \mathbb {R}$. In particular, if $f \in H_{2-2k}$, then *f* has a Fourier expansion of the shape

2.7 $$\begin{aligned} f(\tau ) = \sum _{n \gg -\infty } c_f^+(n) q^n + \sum _{n < 0} c_f^-(n)\Gamma (2k-1,-4\pi nv)q^n. \end{aligned}$$

By [2, Proposition 5.15 iii), iv)], the flipping operator satisfies

2.8 $$\begin{aligned} \xi _{2-2k}({\mathfrak {F}}_{2-2k}(f))&= \frac{(4\pi )^{2k-1}}{(2k-2)!} {\mathcal {D}}^{2k-1}(f), \end{aligned}$$

2.9 $$\begin{aligned} {\mathcal {D}}^{2k-1}({\mathfrak {F}}_{2-2k}(f))&= \frac{(2k-2)!}{(4\pi )^{2k-1}} \xi _{2-2k}(f). \end{aligned}$$

In other words, the flipping operator “switches” the holomorphic and non-holomorphic part in the Fourier expansion of a harmonic Maass form of manageable growth.

### Locally harmonic Maass forms

Let $D \in \mathbb {N}$ be a non-square discriminant and define

2.10 $$\begin{aligned} E_D {:}{=}\bigcup _{Q \in {\mathcal {Q}}_D} \left\{ \tau \in \mathbb {H}:Q_{\tau } = 0 \right\} . \end{aligned}$$

We recall the following definition from [3, Section 2].

#### Definition 2.3

A function $f:\mathbb {H}\rightarrow \mathbb {C}$ is called a *locally harmonic Maass form of weight* $2-2k$ with exceptional set $E_D$, if it satisfies the following conditions:

1. We have $f\vert _{2-2k}\gamma =f$ for every $\gamma \in \Gamma$.
2. For all $\tau \in \mathbb {H}\setminus E_D$, there exists a neighborhood of $\tau$, in which *f* is real-analytic and $\Delta _{2-2k}(f)(\tau )=0$.
3. For every $\tau \in E_D$, we have that
  $$\begin{aligned} f(\tau ) = \frac{1}{2}\lim _{\varepsilon \rightarrow 0^+} (f(\tau +i\varepsilon )+f(\tau -i\varepsilon )). \end{aligned}$$
4. The function *f* exhibits at most polynomial growth towards $i\infty$.

According to [3, Theorem 1.1], ${\mathcal {F}}_{1-k,D}$ from (1.3) is such a locally harmonic Maass form with exceptional set $E_D$. A key property of ${\mathcal {F}}_{1-k,D}$ is that it admits a certain splitting, which might be viewed as a generalization of the Fourier expansion (2.6). To describe this, we define the *holomorphic* and *non-holomorphic Eichler integrals* [11, Section 2] of a cusp form $f(\tau )=\sum _{n\ge 1} c_f(n) q^n\in S_{2k}$ by

$$\begin{aligned} {\mathcal {E}}_f(\tau ) {:}{=}\sum _{n\ge 1} \frac{c_f(n)}{n^{2k-1}}q^n, \qquad f^*(\tau ) {:}{=}(2i)^{1-2k}\!\int _{-{\overline{\tau }}}^{i\infty } f^c(z)(z+\tau )^{2k-2} dz, \end{aligned}$$

where $f^c(\tau ){:}{=}\overline{f(-{\overline{\tau }})}$. We have

$$\begin{aligned} \xi _{2-2k} \left( f^{*}\right) = f, \quad {\mathcal {D}}^{2k-1} \left( f^{*}\right) = 0, \quad \xi _{2-2k} \left( {\mathcal {E}}_{f}\right) = 0, \quad {\mathcal {D}}^{2k-1} \left( {\mathcal {E}}_{f}\right) = f. \end{aligned}$$

Note that both ${\mathcal {E}}_f$ and $f^*$ are real-analytic on $\mathbb {H}$.

#### Lemma 2.4

[ [3, Theorem 7.1]] Let ${\mathcal {C}} \subseteq \mathbb {H}\setminus E_D$ be a connected component and $\tau \in {\mathcal {C}}$. Let

$$\begin{aligned} c_\infty (k) {:}{=}\frac{1}{2^{2k-2}(2k-1)} \sum _{a\ge 1} \sum _{\begin{array}{c} 0\le b<2a\\ b^2\equiv D\pmod {4a} \end{array}} \frac{1}{a^{k}}, \end{aligned}$$

and define the local polynomial by

2.11 $$\begin{aligned} P_{{\mathcal {C}}}(\tau ) {:}{=}-\frac{c_\infty (k)}{\left( {\begin{array}{c}2k-2\\ k-1\end{array}}\right) } + (-1)^k 2^{3-2k} D^{\frac{1}{2}-k} \sum _{\begin{array}{c} Q=[a,b,c]\in {\mathcal {Q}}_D\\ a<0<Q_\tau \end{array}} Q(\tau ,1)^{k-1}. \end{aligned}$$

Then, we have

$$\begin{aligned} {\mathcal {F}}_{1-k,D}(\tau ) = D^{\frac{1}{2}-k} f_{k,D}^*(\tau ) - D^{\frac{1}{2}-k} \frac{(2k-2)!}{(4\pi )^{2k-1}} {\mathcal {E}}_{f_{k,D}}(\tau ) + P_{{\mathcal {C}}}(\tau ). \end{aligned}$$

#### [Style3 Style3]Remark

The constant $c_\infty (k)$ can be evaluated using [22, Proposition 3].

Note that the splitting in Lemma 2.4 resembles the Fourier expansion (2.6) of a harmonic Maass form of manageable growth with $c_f^-(0)v^{k-1}$ replaced by the local polynomial $P_{{\mathcal {C}}}$.

Inspecting a modular integral introduced by Parson [21] in 1993, the second author [19] introduced the function

$$\begin{aligned} g_{k+1,D}(\tau ) {:}{=}\sum _{Q \in {\mathcal {Q}}} \frac{{{\,\textrm{sgn}\,}}\left( Q_{\tau }\right) }{Q(\tau ,1)^{k+1}}, \qquad \tau \not \in E_D, \end{aligned}$$

which can be thought of as an “odd” local variant of $f_{k,D}$ as well as a positive weight analog of ${\mathcal {F}}_{1-k,D}$, because it exhibits singularities on the set $E_D$ too. The function $g_{k+1,D}$ satisfies the conditions from Definition 2.3 by [19, Theorem 1.1]. Moreover, this function appeared in [5] while investigating certain functions introduced by Knopp [15] in 1990. We conclude by citing the following results from [5].

#### Lemma 2.5

1. Both Eichler integrals ${\mathcal {E}}_{g_{k+1,D}}$ and $g_{k+1,D}^{*}$ may be defined on $E_D$.
2. The function ${\mathcal {G}}_{-k,D}$ is a locally harmonic Maass form of weight $-2k$ with continuously but not differentially removable singularities on $E_D$.
3. If $\tau \in \mathbb {H}\setminus E_D$, then we have
  $$\begin{aligned} {\mathcal {G}}_{-k,D}(\tau ) = \pi D^{k+\frac{1}{2}}c_{\infty }(k+1) - \frac{D^{k+\frac{1}{2}}(2k)!}{(4\pi )^{2k+1}}{\mathcal {E}}_{g_{k+1,D}}(\tau ) + D^{k+\frac{1}{2}} g_{k+1,D}^{*}(\tau ). \end{aligned}$$
  In particular, we have
  $$\begin{aligned} {\mathcal {D}}^{2k+1}\left( {\mathcal {G}}_{-k,D}(\tau )\right)&= - \frac{D^{k+\frac{1}{2}}(2k)!}{(4\pi )^{2k+1}}g_{k+1,D}(\tau ), \\ \xi _{-2k}\left( {\mathcal {G}}_{-k,D}(\tau )\right)&= D^{k+\frac{1}{2}}g_{k+1,D}(\tau ). \end{aligned}$$

#### Proof

The first part is the remark after [5, Proposition 4.4]. The second part is [5, Theorem 1.3 (1)]. The third part is [5, Theorem 1.3 (2)] and [5, Proposition 5.2 (1) and (2)]. $\square$

## Proof of Theorem 1.1 and Corollary 1.2

In this section we prove our main result and our corollary.

### Proof of Theorem 1.1

We use Lemma 2.4 and define the real-analytic function

$$\begin{aligned} {\mathbb {F}} {:}{=}D^{\frac{1}{2}-k} f_{k,D}^* - D^{\frac{1}{2}-k} \frac{(2k-2)!}{(4\pi )^{2k-1}} {\mathcal {E}}_{f_{k,D}}. \end{aligned}$$

Using (2.8), (2.9), and (1.4) gives that

$$\begin{aligned} \xi _{2-2k}({\mathfrak {F}}_{2-2k}({\mathbb {F}})) = -\xi _{2-2k}({\mathbb {F}}), \quad {\mathcal {D}}^{2k-1}({\mathfrak {F}}_{2-2k}({\mathbb {F}})) = -{\mathcal {D}}^{2k-1}({\mathbb {F}}). \end{aligned}$$

Since both terms of ${\mathbb {F}}$ decay towards $i\infty$ ($f_{k,D}$ is a cusp form), this shows that

$$\begin{aligned} {\mathfrak {F}}_{2-2k}({\mathbb {F}}) = -{\mathbb {F}}. \end{aligned}$$

So we are left to prove that ${\mathfrak {F}}_{2-2k}(P_{{\mathcal {C}}})=-P_{{\mathcal {C}}}$. By definition of $P_{{\mathcal {C}}}$ in (2.11), it suffices to show that

3.1 $$\begin{aligned} {\mathfrak {F}}_{2-2k}(1)&= -1,\end{aligned}$$

3.2 $$\begin{aligned} {\mathfrak {F}}_{2-2k} (p)&= -p, \end{aligned}$$

where

$$\begin{aligned} p(\tau ) {:}{=}(-1)^k 2^{3-2k} D^{\frac{1}{2}-k} \sum _{\begin{array}{c} Q=[a,b,c]\in {\mathcal {Q}}_D\\ a<0<Q_\tau \end{array}} Q(\tau ,1)^{k-1}. \end{aligned}$$

We start by proving (3.1), which is equivalent to showing that

$$\begin{aligned} R_{2-2k}^{2k-2}(1) = (2k-2)! v^{2-2k}. \end{aligned}$$

This follows directly using (2.5).

We next prove (3.2). Since $\tau \not \in E_D$ and *D* a non-square, we may rewrite

$$\begin{aligned} p(\tau ) = (-1)^k 2^{1-2k} D^{\frac{1}{2}-k} \sum _{Q=[a,b,c]\in {\mathcal {Q}}_D} \left( 1-{{\,\textrm{sgn}\,}}(a)\right) \bigg ({{\,\textrm{sgn}\,}}\left( Q_\tau \right) +1\bigg )Q(\tau ,1)^{k-1}. \end{aligned}$$

Next observe that

$$\begin{aligned} R_{\kappa } \left( \bigg ({{\,\textrm{sgn}\,}}\left( Q_\tau \right) +1\bigg )Q(\tau ,1)^{k-1}\right) = \bigg ({{\,\textrm{sgn}\,}}\left( Q_\tau \right) +1\bigg ) R_{\kappa } \left( Q(\tau ,1)^{k-1}\right) \end{aligned}$$

for $\kappa \in \mathbb {Z}$, because $\frac{d}{d\tau } ({{\,\textrm{sgn}\,}}(Q_\tau )+1) = 0$. Thus, (3.2) follows if we show that

$$\begin{aligned} {\mathfrak {F}}_{2-2k}\!\left( Q(\tau ,1)^{k-1}\right) = -Q(\tau ,1)^{k-1}. \end{aligned}$$

This is equivalent to

3.3 $$\begin{aligned} R_{2-2k}^{2k-2}\!\left( Q(\tau ,1)^{k-1}\right) = \frac{(2k-2)!}{v^{2k-2}} Q({\overline{\tau }},1)^{k-1}. \end{aligned}$$

To prove this note that there exists $A\in \textrm{SL}_2(\mathbb {R})$ (see [3, Lemma 3.1]) such that

3.4 $$\begin{aligned} \tau \vert _{-2} A = -\frac{Q(\tau ,1)}{\sqrt{D}}. \end{aligned}$$

By the definition of the slash operator, (3.4) is equivalent to

$$\begin{aligned} \tau ^{k-1} \big |_{2-2k} A = \frac{(-1)^{k+1}}{D^\frac{k-1}{2}} Q(\tau ,1)^{k-1}. \end{aligned}$$

Using (2.4) repeatedly, we infer

$$\begin{aligned} R_{2-2k}^{2k-2}\left( Q(\tau ,1)^{k-1}\right) = (-1)^{k+1} D^\frac{k-1}{2} R_{2-2k}^{2k-2}\left( \tau ^{k-1}\right) \Big |_{2k-2}A. \end{aligned}$$

To prove (3.3), and hence (3.2) as well, we next claim that it suffices to verify

3.5 $$\begin{aligned} R_{2-2k}^{2k-2}\left( \tau ^{k-1}\right) = (2k-2)! \left( \frac{{\overline{\tau }}}{v^2}\right) ^{k-1}. \end{aligned}$$

Indeed, assuming (3.5) gives

$$\begin{aligned} R_{2-2k}^{2k-2}\left( Q(\tau ,1)^{k-1}\right) = (-1)^{k+1} D^\frac{k-1}{2} (2k-2)! \left( \frac{{\overline{\tau }}^{k-1}}{v^{2k-2}}\right) \bigg |_{2k-2} A. \end{aligned}$$

Rewriting yields

$$\begin{aligned} \left( \frac{{\overline{\tau }}^{k-1}}{v^{2k-2}}\right) \bigg |_{2k-2} A = \frac{(-1)^{k+1}}{D^\frac{k-1}{2}} v^{2-2k} Q({\overline{\tau }},1)^{k-1}. \end{aligned}$$

This directly gives (3.3).

Hence, we are left to prove (3.5). By (2.5), we have

$$\begin{aligned} R_{2-2k}^{2k-2}\left( \tau ^{k-1}\right) = \sum _{r=0}^{2k-2} (-1)^r \left( {\begin{array}{c}2k-2\\ r\end{array}}\right) (2-2k+r)_{2k-2-r} v^{r-2k+2} (4\pi )^r {\mathcal {D}}^r\left( \tau ^{k-1}\right) . \end{aligned}$$

If $r>k-1$, then ${\mathcal {D}}^r(\tau ^{k-1}) = 0$. If $r\le k-1$, then

$$\begin{aligned} {\mathcal {D}}^r\left( \tau ^{k-1}\right) = \frac{1}{(2\pi i)^r} \frac{(k-1)!}{(k-r-1)!} \tau ^{k-1-r}. \end{aligned}$$

Moreover

$$\begin{aligned} (2-2k+r)_{2k-2-r} = (-1)^r (2k-r-2)!. \end{aligned}$$

We then verify (3.5) by obtaining

$$\begin{aligned} R_{2-2k}^{2k-2}\!\left( \tau ^{k-1}\right)&= (2k\!-\!2)! \!\left( \frac{\tau }{v^2}\right) ^{\!k-1} \sum _{r=0}^{k-1} \frac{(k\!-\!1)!}{r!(k\!-\!1\!-\!r)!} \!\left( \frac{-2iv}{\tau }\right) ^{\!r} = (2k\!-\!2)! \!\left( \frac{{\overline{\tau }}}{v^2}\right) ^{\!k-1}. \end{aligned}$$

This completes the proof. $\square$

It remains to prove Corollary 1.2.

### Proof of Corollary 1.2

Let $\tau \in \mathbb {H}\setminus E_D$ and define

$$\begin{aligned} \mathbb {G}(\tau ) {:}{=}D^{k+\frac{1}{2}} g_{k+1,D}^{*}(\tau ) - \frac{D^{k+\frac{1}{2}}(2k)!}{(4\pi )^{2k+1}}{\mathcal {E}}_{g_{k+1,D}}(\tau ). \end{aligned}$$

Using (2.8), (2.9), and Lemma 2.5 (3) gives that

$$\begin{aligned} \xi _{-2k}\left( {\mathfrak {F}}_{-2k}({\mathbb {G}}(\tau ))\right) = -\xi _{-2k}({\mathbb {G}}(\tau )), \quad {\mathcal {D}}^{2k+1}({\mathfrak {F}}_{-2k}({\mathbb {G}}(\tau ))) = -{\mathcal {D}}^{2k+1}({\mathbb {G}}(\tau )). \end{aligned}$$

According to [19, Theorem 1.1 (ii)] (or [5, Proposition 4.1]), $g_{k+1,D}$ decays towards $i\infty$ like a cusp form. Thus, both terms in ${\mathbb {G}}$ decay towards $i\infty$ as well. Hence, we infer that

$$\begin{aligned} {\mathfrak {F}}_{-2k}({\mathbb {G}}(\tau )) = -{\mathbb {G}}(\tau ). \end{aligned}$$

Letting $k \mapsto k+1$ in (3.1) and using the splitting in Lemma 2.5 (3) completes the proof. $\square$

## Data Availability

No datasets were generated or analysed during the current study.

## Footnotes

1 The set $E_D$ is a union of geodesics associated to $Q \in {\mathcal {Q}}_D$.

2 The reader should be aware that their terminology refers to our harmonic Maass forms of manageable growth as “*weak* Maass forms.”
